# The Grand Challenges for Evolutionary Psychology: Survival Challenges for a Discipline

**DOI:** 10.3389/fpsyg.2017.01727

**Published:** 2017-09-27

**Authors:** Peter K. Jonason

**Affiliations:** School of Social Sciences and Psychology, Western Sydney University, Penrith, NSW, Australia

**Keywords:** evolutionary psychology, research methods, sex differences, life history theory, replication

As defined by some of the founders of the field, Barkow et al. (1992), “evolutionary psychology is simply psychology that is informed by the additional knowledge that evolutionary biology has to offer, in the expectation that understanding the process that designed the human mind will advance the discovery of its architecture.” In the field of biology, there is no (with the exceptions of “creationist” or “intelligent design” research) line drawn between evolutionary and non-evolutionary approaches because “nothing in biology makes sense except in the light of evolution” (Dobzhansky, 1973). Like in biology, evolutionary psychologists are often interested in understanding the ultimate adaptations that characterize organisms and account for variance in their behavior. Adaptations are evolved solutions (e.g., color vision for seeing ripe fruit) for specific problems that contribute directly or indirectly to successful reproduction. Adaptations have three characteristics. They occur reliably in a species (e.g., cross-culturally), they are effective at solving adaptive tasks, and they impose reasonable costs on the person.

If one assumes, like evolutionary psychologists do, that psychological systems are biological and physical (i.e., no ethereal concept of mind) in nature, evolutionary models must apply to the brain and its *sequalae*. However, since at least Descartes and, perhaps as far back as Plato, a mind-body dualism has existed whereby the mind (i.e., *psyche*) has been treated as distinct from the body and there is a tendency to treat humans as distinct from “animals” in some form of implicit anthropocentrism which has led to psychological theories generally being developed in parallel deafness to biological theories (Jonason and Dane, 2014). However, such dualism is problematic as it is (1) less parsimonious than monism and (2) creates untestable hypotheses. Evolutionary psychology is a field that tries to reconcile this problem to integrate the study of human behavior and mental mechanisms with the larger biological literature through interdisciplinary means. It tries to treat humans as just another species and assumes that the models researchers use to understand species from tardigrades to blue whales can be used to explain human variability and outcomes.

Adopting an evolutionary framework to the study of human behavior and psychology has been incredibly fruitful. I cannot hope to do it justice here but, instead, I will highlight some of the major areas that evolutionary psychology has provided novel insights. Even work using genetic or hormonal assays, on their own, are merely descriptive in nature indicating that, for instance, being high in sensation-seeking is heritable (e.g., Derringer et al., 2010). Such information tells researchers and people nothing about the ultimate “why” questions that are at the heart of reductionist models of science; evolutionary psychology is reductionistic in nature^1^. First, psychology, since its inception, has been about understanding why individuals differ from one another. However, the field of personality psychology has been stuck in an atheoretical rut after questionable first attempts were made by Freud to generate grand theories of personality. For decades, the field has spent time on descriptive, exploratory, and measurement tasks. Work by evolutionary informed personality researchers has shown how one can derive a new way of understanding personality variation—even some of our darkest and most undesirable traits—as adaptive solutions to contextual conditions to solve mating and survival tasks (e.g., Jonason et al., 2009). Second, psychologists have often been motivated to help other people, but how the field thinks about psychopathologies is particularly atheoretical. Researchers have shown how understanding the evolutionary functions of apparent disorders like depression or obsessive-compulsive disorders may only appear maladaptive given a mismatch between the contexts or function of those traits and the context one finds themselves in or the goals one has chosen to pursue (e.g., Del Giudice, 2014). And last, mate choice and mating strategies are fundamental to the inclusive fitness of all organisms, therefore, unsurprisingly, it is an area under heavy research by evolutionary psychologists. Researchers (for review see, Buss, 2003) have made tremendous strides in tearing down walls of misconceptions about the objective nature of attractiveness (e.g., lumbar shape in women, waist-to-hip ratio, facial symmetry), sex differences in mate choice, tactics to keep and leave one's lovers, the adaptive nature of cheating and other forms of casual sex, and the role of ovulatory hormones in women to influence mate choice (among other things).

Despite this rather simple premise and extensive/impressive research, the field is mired in controversy (e.g., Satoshi Kanazawa's redacted *Psychology Today* blog of racial differences in attractiveness), misunderstandings (Buss and Schmitt, 2011), criticisms (Jonason and Schmitt, 2016), and even accusations of sexism (Schmitt, 2015). There is a constant need to justify the place and utility of evolutionary models of human behavior at the proverbial table of psychological research and defend itself against questions of its scientific legitimacy (e.g., evolutionary psychology is composed of “just so stories”) and evidentiary power (Schmitt, 2008; Li and Meltzer, 2015). There is even some indication of direct bias against evolutionary models as in some cases, the burden of proof for publishing papers that appear to refute evolutionary models appears lower than the burden of proof for those advancing evolutionary models (see Schmitt, 2012, 2014; Schmitt et al., 2012). These represent existential threats to evolutionary psychology and warrant more direct attention.

How might the field begin to approach addressing these issues? There are a few types of submissions that will help in this effort. First, theory/commentary papers that respond to papers published elsewhere along with more expansive theory papers that better articulate the utility of evolutionary models. For example, researchers might publish a paper in another journal that claims to refute evolutionary predictions. A response in the form of a note or commentary might be warranted to lay out why the target article does not actually refute evolutionary models on theoretical or methodological grounds. Alternatively, notes or commentary that further develop theoretical issues are warranted and even present modern updates of “old” theories like sexual strategies theory (Buss and Schmitt, 1993).

Second, replications of “big” papers in evolutionary psychology are especially warranted. As researchers and lay-people have observed in the last five years, the field of psychology has gone through a crisis of faith. Many of the most famous findings in psychology at large have been cast into doubt or even downright refuted (e.g., facial feedback hypothesis; Buck, 1980; Protzko and Schooler, 2017). As far as I can tell, no concerted effort has been expended to determine if key papers in evolutionary psychology can be replicated. For instance, papers on the card selection task (Cosmides, 1989) or fears of snakes and spiders (Öhman, 2009) could be directly replicated to test the “replicability” of evolutionary psychology. Such projects are probably a good avenue for honors students and student projects and can written up in a rather efficient manner.

Third, while direct replications of “big” papers in evolutionary psychology are useful, extending this research is warranted as well. This allows us to test the boundary conditions of the findings as well as the robustness to, for example, methodological and sampling differences. For instance, testing the cross-cultural robustness to life history models of personality (Jonason et al., 2013), disgust responses (Tybur et al., 2009), or perceptual illusions (Jackson and Willey, 2011). Indeed, given the recent realization that many prior studies may have been underpowered, improving the methods and sample size of such papers is especially appealing. Such projects might be well-suited as quick publications for the more experienced researchers or a good project for Masters level students.

Fourth, there is a long tradition in the legal profession. When two parties disagree on something, they engage in an “adversarial” process. Each party puts forth their argument and evidence in hopes of testing which holds more weight. In contrast, to the legal profession, however, science has a different burden of proof. That burden of proof is based on who has the data that best fits its model and, ideally, refutes alternative models. As such, another way to redress the existential threats to evolutionary psychology is to engage in “adversarial” papers whereby researchers pit two or more psychological theories in accounting for phenomena against each other. Importantly, papers that derive contradictory hypotheses from competing theories are especially useful here as they can simultaneous support one model and refute another (see Li et al., 2013). Indeed, in this case, it is possible evolutionary psychological predictions may fail and this is something that, as a field, researchers must be prepared for and willing to publish. For example, researchers trying to explain sex differences in mate preferences might directly test social role and evolutionary predictions in accounting for sex differences in preferences for physical attractiveness and social status. Such paper might be well-suited for more advanced researchers and even Ph.D. level projects.

Fifth, and last, I propose that meta-science papers (Webster, 2007; Webster et al., 2009; Duffy et al., 2011) might further help in the existential threats facing evolutionary psychology. In hopes of understanding larger trends in evolutionary psychology (e.g., big topics in the field), uncovering bias in citation patterns, and understanding how the field has shifted between topics over the years, meta-science papers are called for. In addition, meta-science papers might help to get a sense of the relative impact of papers in the field, methodological trends, and sampling short-comings in evolutionary psychology relative to other fields may further dispel myths about the field. Such papers have considerable appeal as they can be done by anyone even if one does not have access to new data.

Darwin saw new fields of inquiry opening from his theory of natural selection, one of which is psychology. Modern evolutionary psychologists attempt to answer that call. The field faces many existential threats that warrant direct attention. As such, I consider these to be the grand challenge of evolutionary psychology now and welcome papers that attempt to answers those challenges.

## Ethics statement

This is a review paper requested as part of the journals' editorial policies.

## Author contributions

PJ is solely responsible for the content of this manuscript.

### Conflict of interest statement

The author declares that the research was conducted in the absence of any commercial or financial relationships that could be construed as a potential conflict of interest.

## Footnotes

^1^It is possible that one of the reasons the field is so often opposed with such intensity is that it is reductionistic.
